# Surgery under fire: war injuries and the consequences of delayed care in Ethiopia’s Tigray region

**DOI:** 10.1186/s13031-026-00853-7

**Published:** 2026-10-21

**Authors:** Anne Klausing, Walter Bruchhausen, Jan Wynands

**Affiliations:** 1 Clinic and Polyclinic for Oral, Maxillofacial, and Plastic Facial Surgery, University Hospital Bonn, University of Bonn, Venusberg-Campus 1, Bonn, 53127 Germany; 2 Section Global Health, Institute of Hygiene and Public Health, University Hospital Bonn, University of Bonn, Bonn, Germany; 3 Clinic for Dermato-Oncology and Phlebology, University Hospital Bonn, University of Bonn, Bonn, Germany

**Keywords:** Tigray, Ethiopia, Armed conflict, War-related trauma, Bullet injuries, Blast injuries, Delayed surgical care, Peripheral nerve injury, Reconstructive surgery, Disability

## Abstract

**Background:**

The armed conflict in Ethiopia, particularly in the Tigray region, has caused a substantial burden of war-related injuries in the context of a fragile and disrupted health system. Restricted access to timely surgical care has resulted in delayed presentation and may contribute to secondary complications, contractures, peripheral nerve dysfunction, and long-term disability. However, the consequences of delayed surgical treatment for conflict-related extremity trauma in active war settings remain insufficiently described. This study aimed to characterize conflict-related injuries in Mek’ele, Ethiopia, with a particular focus on delayed surgical care, peripheral nerve injuries, and reconstructive needs.

**Methods:**

This retrospective observational study was conducted between September and December 2023 at Ayder Comprehensive Specialized Hospital in Mek’ele, the capital city of Ethiopia’s Tigray region. Patients presenting with conflict-related injuries were identified through routine clinical documentation. Epidemiological characteristics, prior surgical interventions, presenting complaints, clinical findings, and diagnoses were recorded. Peripheral nerve injuries and reconstructive challenges were analyzed in detail. Surgical treatment plans were categorized into reconstructive, corrective and functional, tendon and muscle, nerve, scar and contracture, limb or tissue removal, and supportive procedures. Group comparisons were performed using chi-square and Fisher’s exact tests, and logistic regression models were used to estimate relative likelihoods. Statistical significance was set at *p* < 0.05.

**Results:**

A total of 197 conflict-related injuries were analyzed, including 108 bullet injuries (54.8%) and 82 blast injuries (41.6%). Peripheral nerve injuries were identified in 54.3% of patients. Bullet injuries were more strongly associated with movement restriction, functional deficits, and peripheral nerve damage, whereas blast injuries were more frequently linked to fractures, soft tissue defects, pain, and scars or wounds. Blast injuries more often required reconstructive procedures and contracture release, while bullet injuries more frequently required corrective and functional surgery. Because most patients presented months to years after injury, primary nerve repair was rarely feasible, resulting in a high demand for secondary salvage procedures such as tendon transfers and muscle transpositions.

**Conclusions:**

Conflict-related extremity trauma in Tigray is characterized not only by injury mechanism but also by the consequences of delayed access to surgical care. Missed opportunities for early definitive treatment contributed to a high burden of peripheral nerve deficits, contractures, reconstructive needs, and long-term functional impairment. Improving access to acute trauma care, early nerve repair, reconstructive surgery, and rehabilitation is essential to reduce disability in conflict-affected settings.

## Introduction

Armed conflicts continue to escalate worldwide. In 2023, the Heidelberg Institute for International Conflict Research (HIIK) documented a total of 369 conflicts worldwide, representing an increase of ten compared to the previous year. The majority of these were intrastate conflicts (252 cases), with sub-Saharan Africa being the region with the highest number of wars [1]. In the HIIK framework, intrastate conflicts occur within a state, typically between government forces and one or more non-state actors, whereas interstate conflicts occur between two or more states [1]. The armed conflict in Ethiopia’s Tigray region, which forms the background to this study, was classified as an intrastate conflict. Not only do these conflicts result in high mortality rates, but they also contribute to a significant burden of injuries and long-term disabilities among civilians and combatants alike. Therefore, a rise in conflict-related activities may demand the management of mass casualty incidents, emphasizing the need to expand existing skills and understanding of various injury mechanisms [2].

In this manuscript, we use conflict—affected setting to describe an area where ongoing or recent armed conflict has disrupted civilian life and healthcare delivery, and low-resource setting to describe a context with major limitations in staffing, supplies, diagnostics, and referral capacity relevant to trauma care. Conflict zones and active war areas are characterized by a broad spectrum of traumatic injuries, with gunshot and explosive injuries playing a dominant role [2–4]. Gunshot wounds, which have been extensively studied in both military and civilian settings, are primarily caused by high-velocity projectiles that penetrate tissues, leading to both localized and systemic damage [5]. In contrast, explosive devices such as improvised explosive devices (IEDs) and landmines have become increasingly prevalent in modern conflicts, producing complex multi-mechanism injuries [5–8]. Blast injuries involve a range of effects—including shock waves (primary blast), flying fragments (secondary blast), impact and throw trauma (tertiary blast), as well as thermal or toxic effects (quaternary blast) [9, 10].

Studies from previous war zones indicate that blast injuries frequently lead to a combination of soft tissue trauma, fractures, and amputations, often necessitating extensive reconstructive surgery and prolonged rehabilitation. In contrast, gunshot wounds, while often more localized, frequently involve vascular and nerve damage, requiring emergency surgical interventions and functional reconstructive procedures [4]. Despite these clear distinctions in injury patterns, comparative research on the long-term outcomes of gunshot versus blast injuries remains limited, particularly in low-resource conflict settings where access to timely surgical care is restricted. Together, these distinct and evolving injury patterns underscore the need for medical and surgical practitioners to adapt to the complex injury profiles associated with contemporary armed conflict.

Ethiopia, particularly the Tigray region, has been one of the most heavily affected areas by armed conflict in recent years, with significant civilian casualties and an increasing burden of war-related injuries [11]. Since the onset of armed conflict in late 2020, the region has experienced widespread violence and population displacement. Healthcare infrastructure in these regions is often overwhelmed or severely damaged, making the management of conflict-related trauma even more challenging. Beyond their immediate mortality, war-related injuries contribute substantially to long-term disability. Delayed access to definitive surgical treatment, the scarcity of specialized surgeons, and the lack of rehabilitation services further exacerbate the long-term disability burden among survivors [12–14]. Asgedom et al. [11] reported that in the first eight months of the war in Tigray, approximately 13.8 out of every 1000 residents sustained war-related injuries. This figure represents the overall prevalence of war-related civilian injuries and was not disaggregated according to the blast-versus-bullet distinction used in the present study, although the same source indicates that gunshot wounds accounted for approximately two-thirds of reported injuries, followed by heavy artillery shelling. The injury prevalence observed in this study is notably higher compared to similar conflict-affected regions, including Gaza war in 2014 (6.4 per 1000 population), Baghdad (4.6 to 5 per 1000 population), and Libya (7.1 per 1000 population) [11]. Nonetheless, there is a significant lack of empirical evidence regarding the situation in Tigray, with limited documentation on the impact of armed conflict. Specifically, no comprehensive data exists on the prevalence, causes, and consequences of conflict-related injuries among civilians in the region.

One of the most concerning aspects of war-related injuries is the high prevalence of peripheral nerve injuries. When left untreated or managed inadequately, these injuries can lead to severe functional impairments and permanent disability [15, 16]. Despite the known impact of nerve injuries in war zones, there is limited knowledge on how these injuries present in low-resource conflict areas and what surgical interventions are feasible under such conditions.

### Objective

This study aimed to characterise and compare injury patterns, treatment approaches, and surgical management needs of bullet versus blast trauma in an active conflict setting in Mek’ele, Ethiopia, using routinely collected clinical data. Specific objectives were to compare the two injury mechanisms with regard to demographic characteristics, previous surgical procedures, presenting complaints, clinical findings including peripheral nerve injuries, planned surgical treatment, and the time between injury and first surgical assessment as well as to explore how delayed presentation constrained options for primary nerve repair and other definitive surgical interventions. A particular focus was placed on peripheral nerve injuries and reconstructive challenges, given their potential to cause significant functional impairment if not managed promptly. By identifying differences between trauma mechanisms and their surgical consequences, the study sought to improve understanding of injury management in this conflict-affected setting.

## Methods

### Study design and setting

This study was designed as a retrospective, single-centre observational study based on routinely collected clinical data from patients assessed during their first presentation to Ayder Comprehensive Specialized Hospital in Mek’ele, Ethiopia, between September and December 2023. Ayder Comprehensive Specialized Hospital is a tertiary referral and teaching hospital in Mek’ele, the capital city of the Tigray region, and the largest referral centre for specialised care in northern Ethiopia. It serves a regional catchment area of several million people from Tigray and neighbouring regions and provides a wide range of surgical services, including trauma and orthopaedic surgery, general surgery and reconstructive procedures. During and after the armed conflict in Tigray, the hospital continued to operate despite severe constraints in staffing, medical supplies, diagnostic capacity and referral pathways, and remained one of the few facilities able to manage complex war-related injuries in the region.

### Study population and data sources

Data were obtained from routine paper-based clinical documentation (patient charts and ward records) generated during surgical assessment at presentation; no dedicated electronic research registry or hospital IT registry was used for this study. Eligible participants were adult patients with documented conflict-related injuries (caused by bullets, blasts, or other conflict-related mechanisms) who were assessed during the study period. For the comparative analyses, we focused on the two largest groups, bullet and blast injuries. Patients without conflict-related injury, duplicate entries, and follow-up encounters after the first documented presentation within the study period were excluded from the analytical cohort. No formal sample size calculation was performed; instead, all consecutively assessed eligible patients during the four-month study period were included.

### Data collection and variables

As part of routine clinical history-taking at presentation, patients were interviewed by members of the treating clinical team regarding previous surgical procedures related to their injuries and their current complaints. In this context, ‘interviewed’ refers to standard face-to-face clinical history taking rather than a separate research interview process. Demographic and clinical data included age, sex, injury mechanism, number and timing of previous operations related to the injury, and patient-reported current complaints. Symptoms considered included pain, functional deficits (for example foot drop, wrist drop, drooling), movement restrictions, physical deformities and other persistent complaints related to the initial injury. Medical findings and diagnoses were based primarily on routine clinical examination documented by the treating surgeons and other clinicians. Where available and clinically indicated, existing imaging (such as radiographs) and previous operative reports were used to support diagnosis, but no additional research-specific diagnostic tests or laboratory investigations were performed for the purposes of this study.

### Data handling and anonymization

Before analysis, all data were anonymised by removing directly identifying information from the extracted dataset. The anonymised dataset was stored in password-protected files accessible only to the study team. The dataset was checked for internal consistency, completeness and duplicate presentation records before statistical analysis; missing data were not imputed.

### Outcomes and superordinate categories

To enable clinically interpretable group comparisons, we grouped previous surgical procedures, presenting complaints, clinical findings and planned surgical treatment into prespecified superordinate categories developed by the authors on clinical grounds, based on the recorded case mix. Previous procedures were grouped as reconstructive surgeries, corrective and functional surgeries, emergency and trauma interventions, and minor or adjunctive procedures. Presenting complaints were grouped as physical deformities, movement restrictions, functional deficits, fractures, scars and wounds, and pain. Clinical findings were grouped as fractures and malunions, contractures, tendon injuries, peripheral nerve injuries, neuromas, facial and cranial nerve injuries, scars, wounds, acute infection, facial defects and fractures, tooth loss, and amputations. Planned treatment was grouped as reconstructive surgeries, corrective and functional surgeries, limb or tissue removal, tendon and muscle surgeries, nerve surgeries, scar and contracture surgeries, and supportive treatments. These categories were defined pragmatically to reflect recurring patterns in the observed injury spectrum and were not based on a specific theoretical framework.

### Statistical analysis

Categorical group differences between bullet and blast injuries were assessed using chi-square or Fisher’s exact tests, as appropriate. Logistic regression models were used to estimate adjusted associations and age-specific predicted probabilities; model coefficients are presented as odds ratios (ORs) with corresponding 95% confidence intervals (CIs). Statistical significance was defined as *p* < 0.05. Statistical analysis was performed using STATA/SE 16.1 (StataCorp, College Station, TX, USA). The reporting of this observational study was informed by the STROBE guidance for observational studies.

## Results

### Demographics

A total of 197 conflict-related injuries were assessed: 54.8% (*n* = 108) by bullets, 41.6% (*n* = 82) by blasts and 3.6% (*n* = 7) by other means. We focused on the two largest patient groups, those with injuries caused by bullets and blasts. 90.9% (*n* = 179) of all patients were male. The gender distribution within the two injury groups differed statistically significantly: 97.2% of the patients in the bullet group and 81.7% of the patients in the blast group were male (*p* < 0.001). The youngest patient was 18 years old, the oldest was 61 years old. The mean age did not differ significantly between the two major groups (28.3 (± 9.0) years in the bullet group vs. 26.7 (± 9.0) years in the blast group; *p* = 0.23) (Table 1).

**Table 1:** Conflict-related bullet and blast casualties in Mek’ele, Ethiopia from September – December 2023

Patients (*n*)		Bullet	Blast	Pvalue
		108	82	
Age (years), mean (± SD; 95%CI)		28.3 (± 9.0; 26.6–30.0)	26.7 (± 9.0; 24.7–28.7)	0.23
Gender (% male)		97.2	81.7	*< 0.001* ^**^
Previous surgeries (n)	*0*	58	44	0.90
	*1*	41	27	0.54
	*2*	11	12	0.33
	*3*	4	2	0.64
Duration of injury (months), mean (± SD; 95%CI)		24.8 (± 5.0; 23.8–25.8)	23.5 (± 4.2; 22.6–24.4)	0.05*

*/** indicates statistically significant group difference

### Previous surgical procedures

At the time of the survey, a total of 97 patients had undergone at least one operation or surgical procedure as part of their initial hospital presentation due to their injury. This was on average 24.8 (± 5.0; 95%CI 23.8–25.8) months (bullet group) or 23.5 (± 4.2; 95%CI 22.6–24.4) months (blast group) ago (*p* = 0.05) (Table 1).

Surgical procedures performed at initial hospital presentation were summarised using the predefined categories described in the Methods (reconstructive surgeries, corrective and functional surgeries, emergency and trauma interventions, and minor or adjunctive procedures). There were no statistically significant differences in the distribution of these procedures between patients wounded through bullets (total procedures *n* = 115) or blasts (total procedures *n* = 89) (*p* = 0.725) (Table 2). In this cohort, patients with bullet injuries more frequently required emergency interventions and major, invasive procedures, such as limb and tissue removal, although these differences were not statistically significantly (Table 2).

**Table 2:** Operative procedures performed and/or planned on 108 bullet casualties and 82 blast casualties

Procedures performed during initial presentation			Further surgical treatment plan		
Bullet	Blast		Bullet	Blast	
17	13	Reconstructive surgeries	11	19	Reconstructive surgeries (*p* = 0.05*)
18	12	Corrective and functional surgeries	45	27	Corrective and functional surgeries (*p* = 0.05*)
3	1	Limb or tissue removal	2	2	Limb or tissue removal
14	8	Emergency and trauma interventions	41	41	Tendon and muscle surgeries
4	7	Minor or adjunctive procedures	1	4	Nerve surgeries
59	48	None	7	15	Scar and contracture surgeries (*p* = 0.04*)
			32	16	Supportive treatments (*p* = 0.04*)
			1	0	None
**115**	**89**	**Total procedures**	**140**	**124**	**Total procedures**
*p* = 0.725			*p* = 0.011*		

* indicates statistically significant group difference

### Complaints and medical findings on hospital presentation

**Fig. 1:**
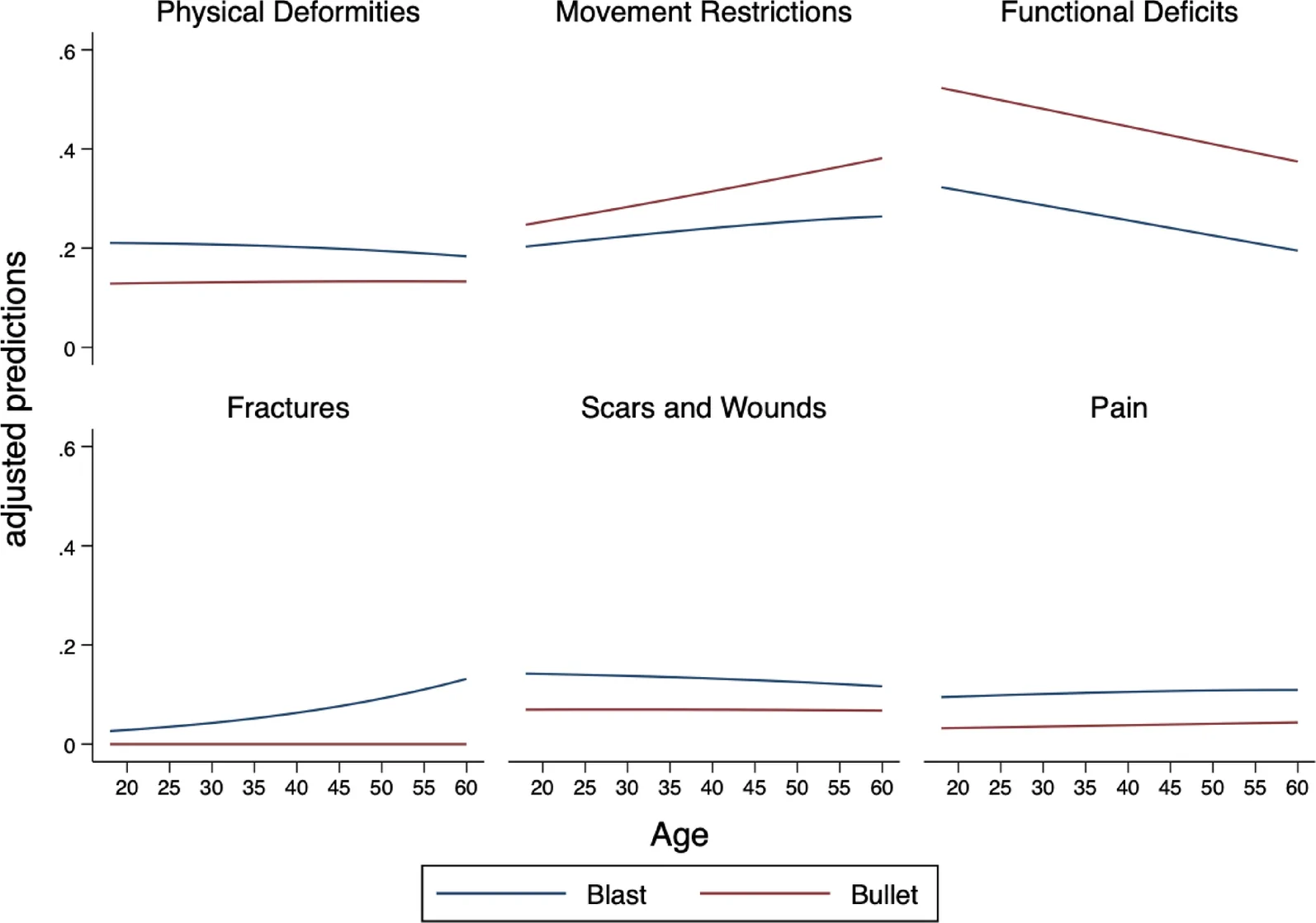
**Age-specific adjusted probabilities of complaints mentioned at presentation depending on trauma mechanism.** The figure shows model-based age-specific adjusted predictions for physical deformities, movement restrictions, functional deficits, fractures, scars and wounds, and pain among patients with conflict-related injuries in Mek’ele, Ethiopia. Predicted probabilities are displayed separately for blast injuries (blue) and bullet injuries (red)

Patient-reported complaints at presentation were analysed using the predefined superordinate categories described in the Methods section (physical deformities, movement restrictions, functional deficits, fractures, scars and wounds, and pain). Proportions (n/N, %) with 95% confidence intervals by age group and trauma mechanism are presented in Table 3. Age-specific adjusted probabilities of presenting complaints derived from the regressions models are shown in Fig. 1.

**Table 3:** Crude proportion of patients with each presenting complaint, by age group and type of trauma

Complaint	Age group [years]	Blast *n*/*N* (%) [95% CI]	Bullet *n*/*N* (%) [95% CI]
Physical deformities	18–29	17/64 (26.6%) [17.1; 38.7]	8/75 (10.7%) [5.4; 20.0]
	30–39	3/9 (33.3%) [11.0; 66.9]	3/19 (15.8%) [5.1; 39.3]
	40–49	0/7 (0.0%) [CI n.e.]	2/7 (28.6%) [7.1; 67.6]
	50–60	0/1 (0.0%) [CI n.e.]	1/7 (14.3%) [1.9; 58.4]
Movement restrictions	18–29	14/64 (21.9%) [13.4; 33.7]	21/75 (28.0%) [19.0; 39.2]
	30–39	0/9 (0.0%) [CI n.e.]	4/19 (21.1%) [8.1; 44.7]
	40–49	1/7 (14.3%) [1.9; 58.4]	3/7 (42.9%) [14.3; 77.2]
	50–60	1/1 (100.0%) [CI n.e.]	3/7 (42.9%) [14.3; 77.2]
Functional deficits	18–29	17/64 (26.6%) [17.1; 38.7]	39/75 (52.0%) [40.7; 63.1]
	30–39	4/9 (44.4%) [17.6; 75.0]	9/19 (47.4%) [26.7; 69.0]
	40–49	3/7 (42.9%) [14.3; 77.2]	1/7 (14.3%) [1.9; 58.4]
	50–60	0/1 (0.0%) [CI n.e.]	3/7 (42.9%) [14.3; 77.2]
Fractures	18–29	2/64 (3.1%) [0.8; 11.7]	0/75 (0.0%) [CI n.e.]
	30–39	0/9 (0.0%) [CI n.e.]	0/19 (0.0%) [CI n.e.]
	40–49	0/7 (0.0%) [CI n.e.]	0/7 (0.0%) [CI n.e.]
	50–60	0/1 (0.0%) [CI n.e.]	0/7 (0.0%) [CI n.e.]
Scars and wounds	18–29	10/64 (15.6%) [8.6; 26.8]	5/75 (6.7%) [2.8; 15.1]
	30–39	0/9 (0.0%) [CI n.e.]	2/19 (10.5%) [2.6; 33.9]
	40–49	2/7 (28.6%) [7.1; 67.6]	0/7 (0.0%) [CI n.e.]
	50–60	0/1 (0.0%) [CI n.e.]	0/7 (0.0%) [CI n.e.]
Pain	18–29	4/64 (6.3%) [2.4; 15.6]	2/75 (2.7%) [0.7; 10.1]
	30–39	2/9 (22.2%) [5.6; 58.1]	1/19 (5.3%) [0.7; 29.7]
	40–49	1/7 (14.3%) [1.9; 58.4]	1/7 (14.3%) [1.9; 58.4]
	50–60	0/1 (0.0%) [CI n.e.]	0/7 (0.0%) [CI n.e.]

Values are n/N (%) with 95% confidence intervals; CI n.e. = confidence interval not estimable

The probability of physical deformities, scars and wounds as well as pain remains relatively stable across all age groups. Movement restrictions increase with age, the probability of complaining of movement restrictions due to bullet injuries rises from 24.7% (95%CI 14.5–34.9; *p* < 0.001) at the age of 18 years to 38.1% (95%CI 11.2–65.1; *p* = 0.006) at the age of 60 years. Functional deficits tend to decrease as age progresses, from the age of 18 to the age of 60 years the probability within bullet injuries decreases from 52.3% (95%CI 39.9–64.6; *p* < 0.001) to 37.5% (95%CI 11.4–63.5; *p* = 0.005). Fractures are significantly less common in bullet injuries. The prevalence of fractures slightly increases with age, particularly among patients with blast injuries (2.6% at the age of 18 to 13.2% at the age of 60 years). In summary, our data shows that bullet injuries are more strongly associated with movement restrictions and functional deficits whereas blast injuries are more frequently linked to fractures, scars/wounds and persisting pain (Fig. 1).

**Fig. 2:**
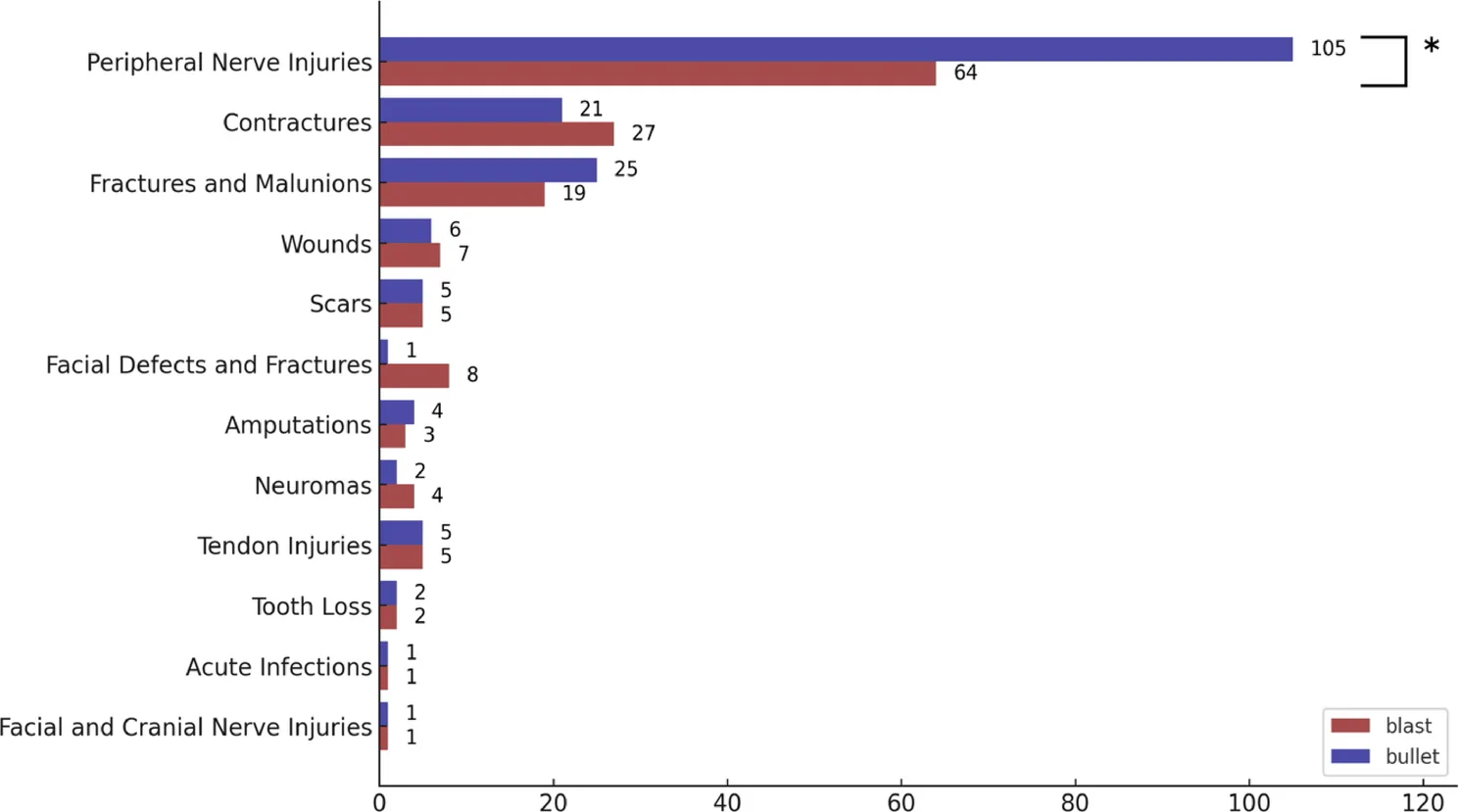
**Frequency of medical findings on presentation depending on trauma mechanism.** The figure shows the frequency of diagnoses among patients with conflict-related injuries, stratified by injury mechanism. Bullet injuries are shown in blue and blast injuries in red. Diagnoses are presented as absolute numbers. Peripheral nerve injuries were the most frequent diagnosis in both groups and were significantly more common in bullet injuries than in blast injuries; * indicates statistically significant group difference

Clinical findings at presentation, grouped as described in the Methods section, showed that peripheral nerve injuries are the most common diagnosis, especially indicating a higher prevalence in bullet-related trauma (*p* = 0.009). Blast injuries, on the other hand, appear to be more associated with contractures, although not statistically significantly (*p* = 0.126) (Fig. 2).

### Peripheral nerve injuries

**Fig. 3:**
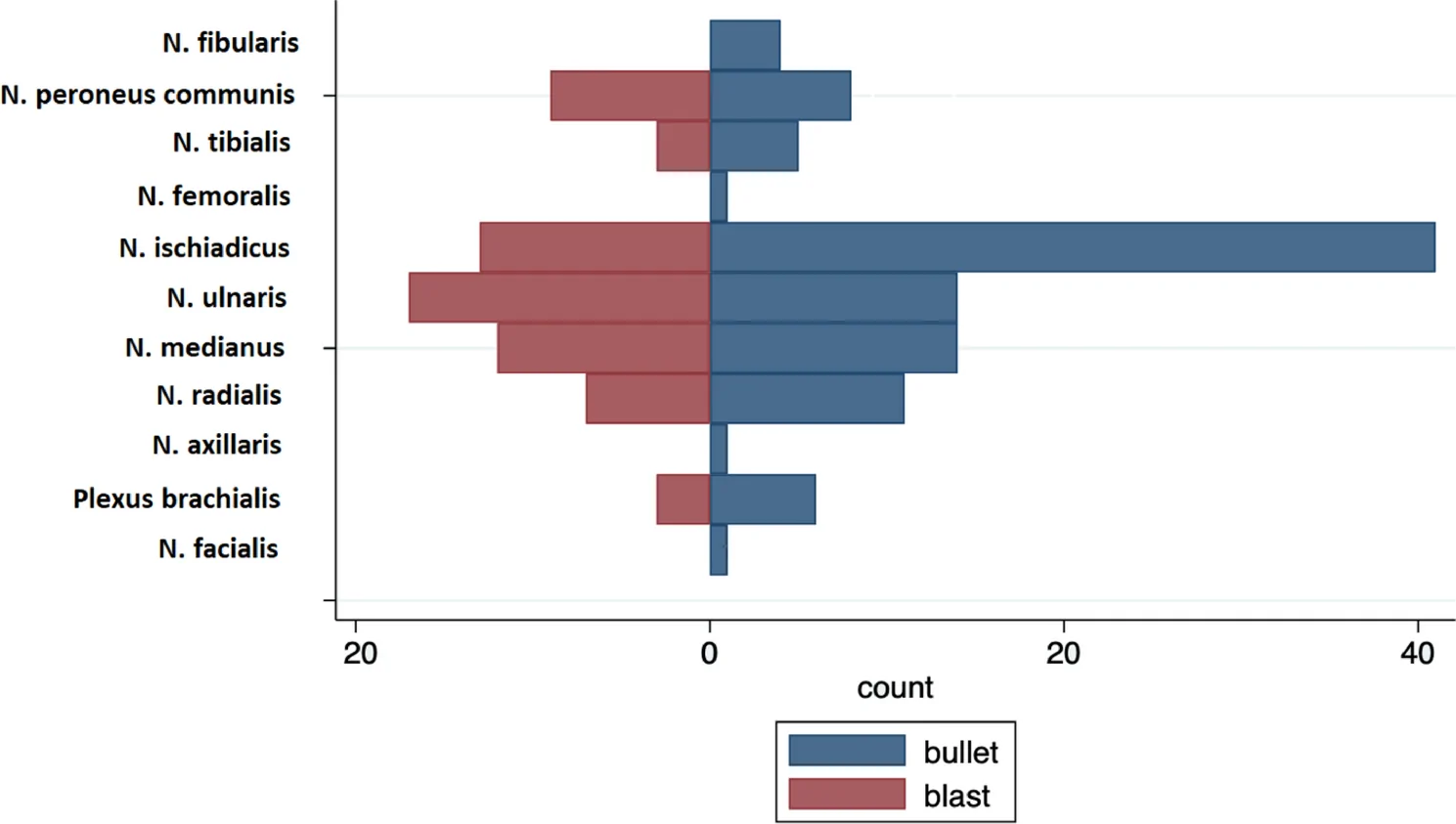
**Distribution of peripheral nerve injuries by affected nerve and injury mechanism.** The figure shows the frequency of affected peripheral nerves among patients with conflict-related injuries, stratified by injury mechanism. Blast injuries are displayed on the left side of the axis in red and bullet injuries on the right side in blue for visual comparison. Absolute counts are shown for each affected nerve. Upper and lower extremity nerve injuries are presented together

More than half of all patients (*n* = 107, 54.3%) presented with injuries to one or more peripheral nerves. Across the entire cohort, a total of 170 injured nerves were observed (Fig. 2). In total, 76 patients (38.6%) sustained injuries to peripheral nerves of the upper extremity (*N. axillaris*,* N. radialis*,* N. medianus*,* N. ulnaris*), while 84 patients (42.6%) had injuries to peripheral nerves of the lower extremity (*N. ischiadicus*,* N. femoralis*,* N. tibialis*,* N. peroneus communis*,* N. fibularis*). The distribution of these peripheral nerve injuries showed distinct and statistically significant differences depending on the injury mechanism (*p* = 0.014) (Fig. 3).

**Fig. 4:**
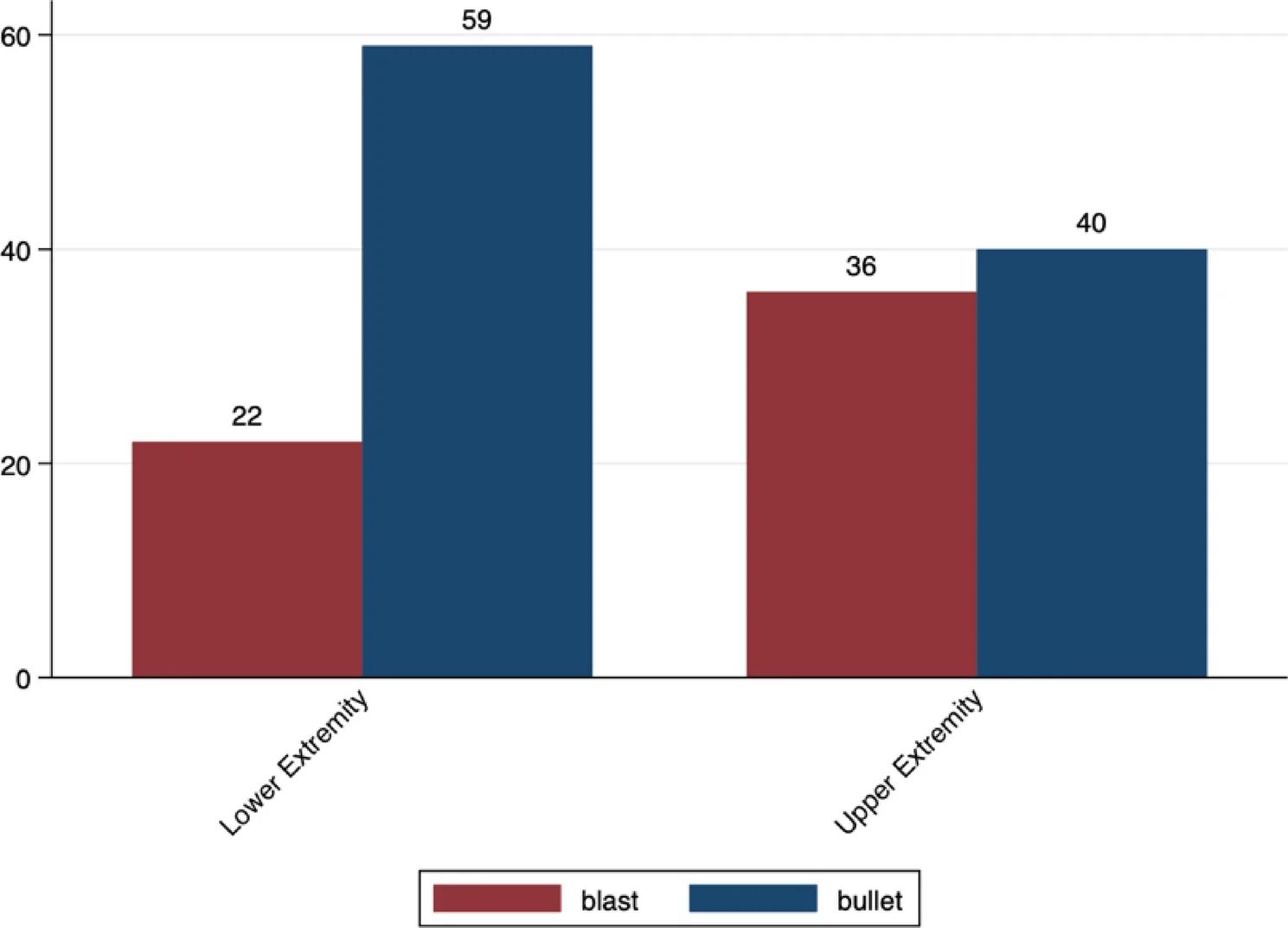
**Comparison of peripheral nerve injury distribution in blast versus bullet injuries.** The distribution of peripheral nerve injuries between the upper and lower extremities differed significantly by injury mechanism, with lower extremity involvement being more frequent in bullet injuries than in blast injuries (*p* = 0.014)

In contrast to blast injuries (*peripheral nerve injuries of the upper extremity: n = 36/107*,* 33.6%; peripheral nerve injuries of the lower extremity: n = 22/107*,* 20.6%*), gunshot injuries (*peripheral nerve injuries of the upper extremity: n = 40/107*,* 37.4%; peripheral nerve injuries of the lower extremity: n = 59/107*,* 55.1%*) exhibited a notable predominance in the lower extremities (Fig. 4).

**Fig. 5:**
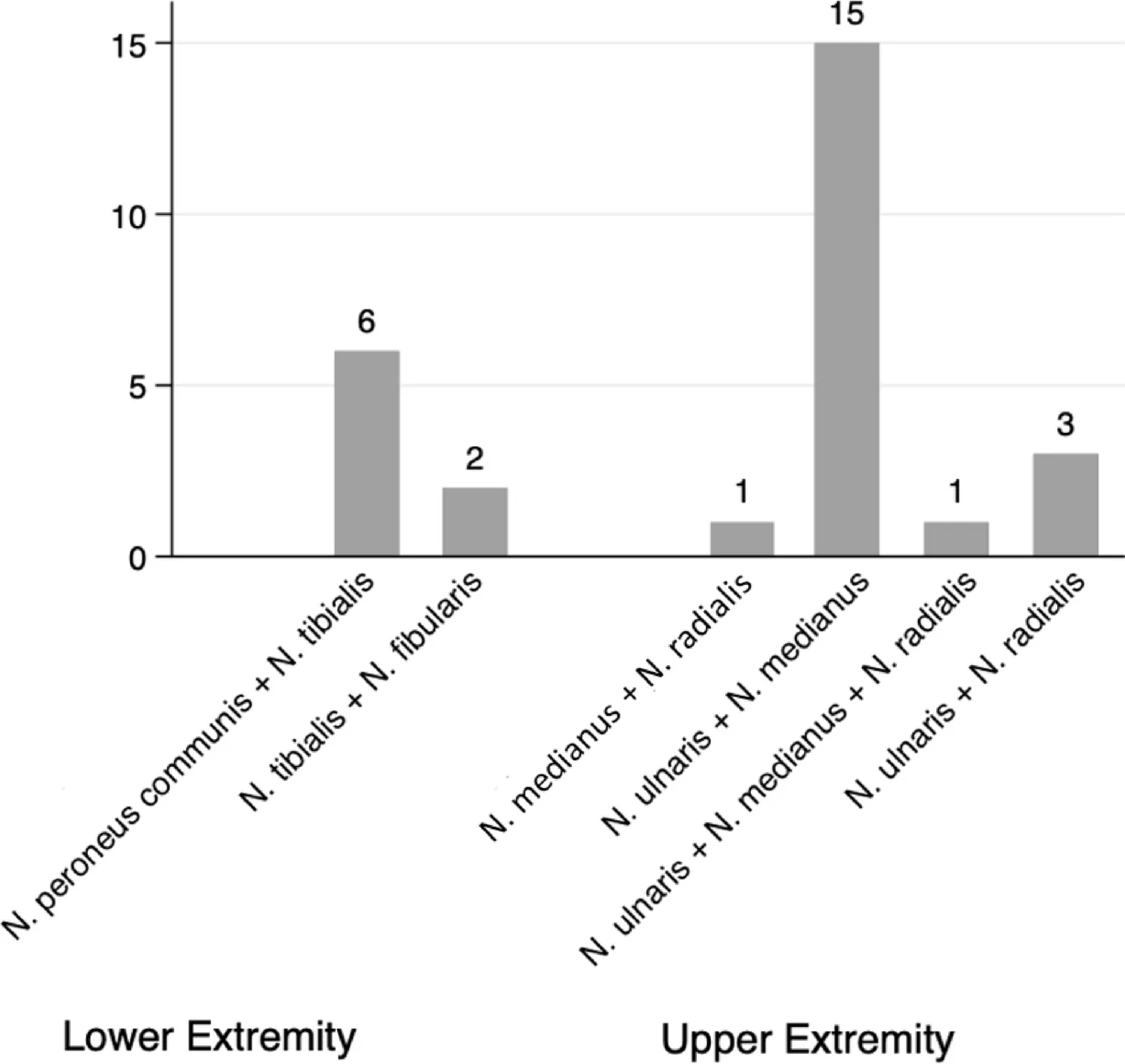
**Distribution of combined peripheral nerve injuries in the upper and lower extremities.** The figure shows the frequency of combined peripheral nerve injuries by anatomical region. Absolute counts are displayed above the bars. Combined ulnar and median nerve injuries were the most frequent pattern in the upper extremity, while combined common peroneal and tibial nerve injuries were most frequent in the lower extremity

Additionally, combined peripheral nerve injuries were observed in 28 patients (26.2%), predominantly affecting the upper extremity nerves (*N. ulnaris + N. medianus (n = 15)*,* N. ulnaris + N. radialis (n = 3)*,* N. medianus + N. radialis (n = 1)*,* N. ulnaris + N. medianus + N. radialis (n = 1)*). Furthermore, in 8 cases, combined peripheral nerve injuries of the lower extremity occurred (*N. peroneus communis + N. tibialis (n = 6)*,* N. tibialis + N. fibularis (n = 2)*) (Fig. 5).

### Surgical treatment plans

**Fig. 6:**
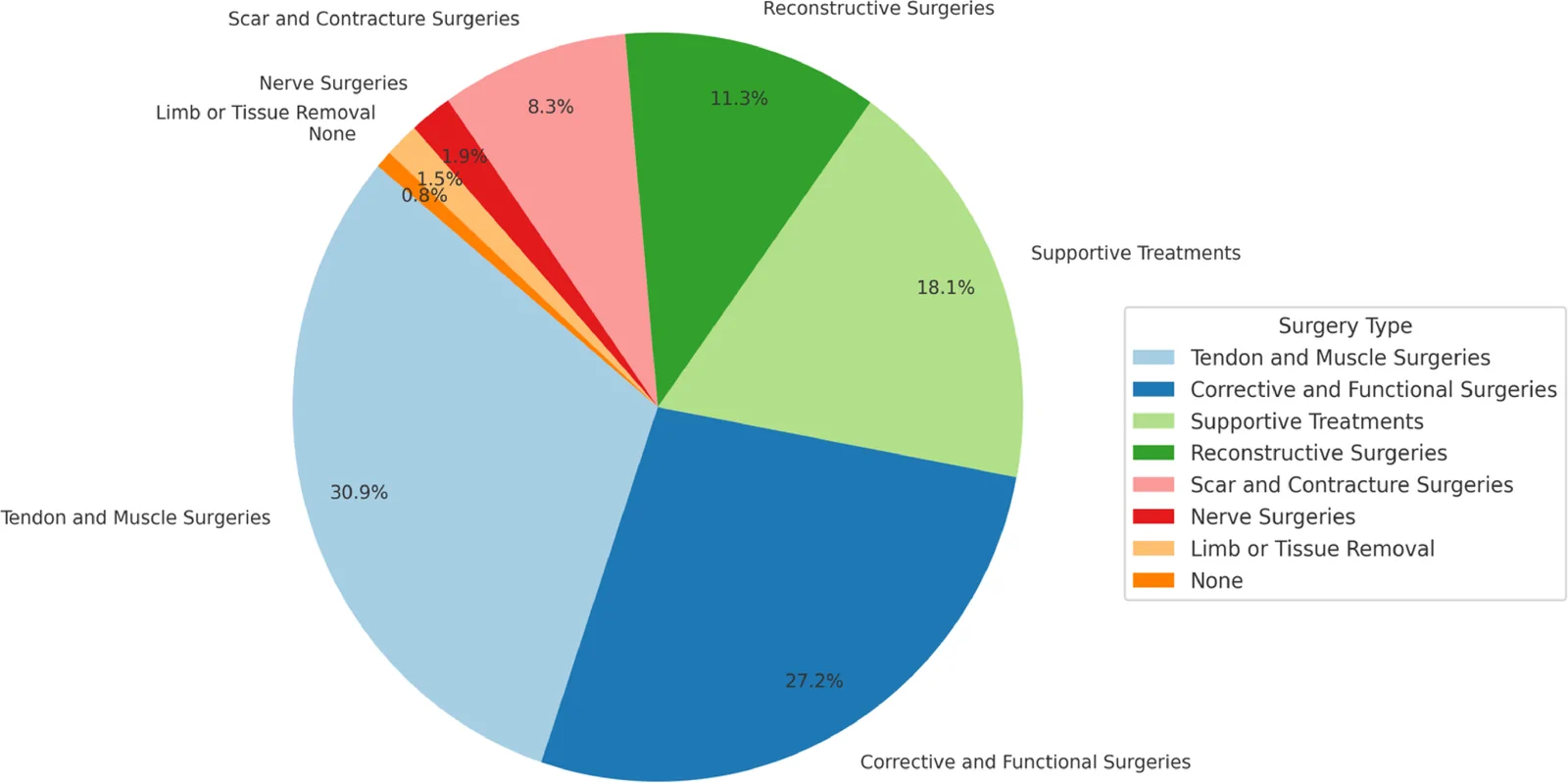
**Distribution of planned surgical treatment categories.** The figure shows the proportional distribution of planned surgical interventions among patients with conflict-related injuries. Tendon and muscle surgeries accounted for the largest proportion of planned procedures, followed by corrective and functional surgeries, supportive treatments, and reconstructive surgeries. Smaller proportions were observed for scar and contracture surgeries, nerve surgeries, limb or tissue removal, and cases without planned surgical intervention. Percentages are shown for each category

Planned surgical treatment was summarised using the predefined categories described in the Methods (reconstructive surgeries, corrective and functional surgeries, limb or tissue removal, tendon and muscle surgeries, nerve surgeries, scar and contracture surgeries, and supportive treatments). The distribution of planned surgical interventions among patients illustrates the broad range of procedures required to manage injuries sustained in conflict settings (Fig. 6). The most common planned interventions were tendon and muscle surgeries (82 cases, 30.9% of all planned surgeries), followed by corrective and functional surgeries (72 cases, 27.2%) and supportive treatments (48 cases, 19.8%). Reconstructive surgeries accounted for 30 cases (11.3%), scar and contracture surgeries were planned in 22 cases (8.3%). Procedures involving limb or tissue removal were rare (4 cases, 1.5%), indicating that while amputation is a recognized outcome of severe trauma, limb salvage and reconstructive efforts are prioritized when possible. Additionally, only 2 patients (0.8%) did not require any surgical intervention, demonstrating that the majority of trauma cases necessitated surgical management.

The overall distribution of planned surgeries underscores the complexity of injury patterns in conflict-related trauma, with a high prevalence of nerve damage and functional impairments.

Analyzed by trauma mechanism, bullet injuries required more corrective and functional surgeries (*n* = 45, 27.2%) compared to blast injuries (*n* = 27, 18.3%) whereas blast injuries showed a higher frequency of reconstructive surgeries (blast: *n* = 19, 15.2%; bullet: *n* = 11, 7.9%) and scar/contracture releases (blast: *n* = 15, 12.0%; bullet: *n* = 7, 5.0%). Both injury groups exhibited an equal number of tendon and muscle surgeries (41 cases each, 30.9%). Only a very small proportion of patients had direct nerve surgery planned at the time of presentation, regardless of the trauma mechanism, suggesting that reconstructive nerve procedures were rarely considered feasible under the prevailing resource constraints.

## Discussion

### Demographics and injury pattern in Mek’ele, Ethiopia

The age distribution of injured individuals in our study aligns with broader demographic trends observed in conflict zones across sub-Saharan Africa [17]. In our cohort, the youngest patient was 18 years old, while the oldest was 61 years old. The mean age did not differ significantly between the two major injury groups, with an average of 28.3 (± 9.0) years in the bullet injury group and 26.7 (± 9.0) years in the blast injury group (*p* = 0.23) (Table 1). This suggests that both types of injuries predominantly affect young adults, which is consistent with findings from other conflict-affected regions in sub-Saharan Africa [18].

Demographic studies indicate that sub-Saharan Africa has the youngest population globally, with a median age of approximately 19.2 years. Over 70% of the region’s population is under the age of 30, reflecting a high proportion of young individuals in both urban and rural areas [17]. Given this demographic structure, it is not surprising that young adults constitute the majority of the injured in conflict settings. Previous research has shown that young men, in particular, are more likely to be directly involved in combat or exposed to violent confrontations, increasing their risk of sustaining injuries [19]. A study by the Human Sciences Research Council highlights the association between traditional constructions of masculinity and young men’s participation in conflict settings, suggesting that societal expectations and gender socialization contribute to their involvement in violence [20].

Furthermore, rural areas, where many young individuals reside, are often among the hardest hit in conflict situations. Research indicates that rural areas in sub-Saharan Africa often bear the brunt of armed conflicts, leading to significant humanitarian crises. For instance, in countries like Ethiopia, Mozambique, Cameroon, and Nigeria, rural communities have been severely affected by violence, resulting in widespread displacement and human rights abuses [21]. Additionally, studies have shown that armed conflicts in sub-Saharan Africa remain predominantly rural, with rural insurgent groups exploiting intercommunal tensions and grievances of marginalized communities [22].

While our study found no significant difference in mean age between the two injury groups, previous literature has reported that different mechanisms of injury can impact age distribution. For example, some studies suggest that explosive devices, often deployed in civilian-populated areas, may result in a slightly younger or more diverse demographic profile compared to gunshot injuries, which are more frequently associated with combatants [23, 24]. However, further research is needed to explore whether similar trends apply specifically to sub-Saharan Africa and to assess potential variations across different conflict settings.

Overall, our study reinforces the importance of considering demographic structures when planning medical interventions and resource allocation in conflict zones. Understanding the age profile of injured patients can help inform triage strategies, rehabilitation planning, and long-term healthcare needs in post-conflict recovery efforts.

Our study examined the adjusted probabilities of various complaints reported at presentation, depending on patient age and trauma mechanism (blast vs. bullet injuries). The findings indicate that bullet injuries are more strongly associated with movement restrictions and functional deficits, whereas blast injuries are more frequently linked to fractures, scars/wounds, and pain (Fig. 1). These results align with previous studies that have documented differences in injury patterns and outcomes between blast and bullet injuries. A retrospective analysis of 333 patients in Somalia found that blast injuries were associated with a higher number of concomitant injuries, a higher Injury Severity Score (ISS), and longer hospital stays, whereas bullet injuries more frequently resulted in open extremity fractures [19]. Another study analyzing 983 civilian blast and ballistic injuries in Afghanistan reported that blast injuries were more frequently associated with amputations, whereas ballistic injuries more often required thoracic drainage procedures. These findings highlight the distinct clinical challenges posed by each injury mechanism [24]. Additionally, a systematic review of injury patterns among civilians in armed conflicts found that blast injuries tend to cause complex, multisystemic trauma requiring extensive medical management [23]. Our findings contribute to the growing understanding of the distinct consequences of blast and bullet injuries, emphasizing the need for tailored treatment and rehabilitation strategies that address the specific challenges associated with each injury mechanism.

### Surgery in an active conflict zone

Our study data shows that in approximately half of all cases involving gunshot and explosion injuries, one or more surgical procedures were required (bullet injuries *n* = 56; 49.1%; blast injuries *n* = 41; 48.2%). At the time of data collection, these surgeries had occurred further in the past for the group of patients with gunshot injuries compared to those treated for explosion injuries (24.8 (± 5.0; 95%CI 23.8–25.8) months vs. 23.5 (± 4.2; 95%CI 22.6–24.4) months; *p* = 0.05) (Table 1). This finding may indicate that injuries caused by bullets require longer and more intensive treatment. However, the actual differences in our study, although statistically significant, are relatively marginal in absolute terms. Considering the procedures performed at initial hospital presentation, it is noticeable that patients with bullet injuries more frequently required emergency interventions and major, invasive procedures, such as limb and tissue removal (Table 2). Several studies confirm that gunshot injuries often require more extensive emergency interventions and invasive procedures compared to blast injuries in conflict settings [19, 25–27]. This trend is particularly evident in sub-Saharan Africa, where gunshot wounds frequently involve severe vascular, bone, and soft tissue damage. For instance, research conducted in Nigeria and Cameroon highlights that patients with gunshot injuries had higher rates of limb fractures and underwent more debridements and amputations compared to those with blast injuries [25, 28]. The focused and localized nature of gunshot trauma often results in direct structural damage, necessitating urgent surgical care, whereas blast injuries typically distribute forces more diffusely, potentially reducing the immediate need for extensive interventions [4]. These findings underscore the need for tailored triage strategies and specialized surgical capacities in conflict zones.

Blast injuries in active conflict are nevertheless gaining increasing significance and have not yet been studied as extensively as bullet injuries. Especially the increased use of improvised explosive devices (IEDs) has particularly drawn attention to the injury patterns associated with blast injuries [4, 5, 29]. The observed gender disparity between the two injury groups, with a significantly higher proportion of males in the bullet injury group compared to the blast injury group (*p* < 0.001), aligns with findings from previous studies [19, 30, 31]. One potential explanation is the direct involvement of men in combat situations, increasing their likelihood of sustaining gunshot wounds. Research from conflict zones, such as a hospital-based study in Gaza, reported that nearly all patients with gunshot injuries were male, emphasizing the strong correlation between combat exposure and firearm-related trauma [30]. In contrast, blast injuries tend to affect a broader demographic, including civilians, as explosions frequently occur in populated areas. Although 81.7% of the blast injured patients are still male, our data shows a significant difference compared to the group of patients injured by bullets (97.2%; *p* < 0.001; Table 1). Reports from the United Nations indicate a rising proportion of women and children among civilian casualties in modern conflicts, suggesting that indiscriminate explosive violence contributes to a more diverse distribution of injuries [31]. Although this study focuses on conflict-related injuries in Tigray, we cannot fully exclude that some cases classified as bullet injuries may have resulted from urban violence or gang-related activity rather than directly from the armed conflict. In many conflict-affected settings, such mechanisms may overlap, and our data do not allow a clear distinction between these contexts. This trend underscores the differing mechanisms of injury exposure and highlights the need for targeted medical and humanitarian responses based on the demographic patterns of trauma in conflict settings.

Additionally, we observed a statistically significant trend indicating that blast injuries more frequently require reconstructive surgeries, such as skin and flap grafts or facial reconstruction surgeries, as well as scar and contracture releases (*p* = 0.05; Table 2). In contrast, bullet injuries were statistically significantly more often associated with planned corrective and functional surgeries, particularly secondary fracture management, including ORIF, arthrodesis, and corrective osteotomy) (*p* = 0.05; Table 2). Comparable studies have reported similar findings, indicating that blast injuries more frequently require extensive reconstructive procedures due to complex soft tissue damage, whereas gunshot injuries often necessitate corrective and functional surgeries to restore structural integrity and mobility [32].

### Delayed access to surgical care and its consequences

One of the most critical findings of this study is that the majority of patients presented for treatment months to years after their initial injury (bullet injuries 24.8 (± 5.0; 23.8–25.8) months; blast injuries 23.5 (± 4.2; 22.6–24.4) months; *p* = 0.05). This delay in access to surgical care highlights significant gaps in medical infrastructure and acute trauma management within conflict settings. Studies from other war-affected regions, including Syria [33] and Somalia [34], have similarly reported that access to timely surgical intervention is often severely restricted, leading to worse functional outcomes.

As a direct consequence, primary nerve repair and reconstruction were no longer feasible at the time of presentation. Instead, the majority of patients required secondary reconstructive procedures, such as tendon transfers and muscle transpositions, which, while providing some functional recovery, are significantly less effective than direct nerve repair. This aligns with findings from Braga Silva et al. (2023), who demonstrated that delayed nerve repairs result in poorer motor and sensory outcomes compared to acute interventions [35].

The lack of acute surgical interventions in this cohort underscores the urgent need for better surgical access during conflicts. In particular, early nerve repair strategies should be prioritized in war-related injuries to prevent the necessity of salvage procedures, which inherently lead to suboptimal recovery and higher rates of permanent disability [16, 36].

### The high burden of peripheral nerve injuries and the need for improved diagnosis

Peripheral nerve injuries were the most common diagnosis in our cohort (*n* = 107, 54.3%), particularly affecting the upper extremities and frequently presenting as combined nerve injuries (e.g., ulnar + median nerve, or common peroneal + tibial nerve). The high prevalence of combined nerve injuries (*n* = 28, 26.2%) suggests that many patients sustained complex, multi-level trauma, which carries a high risk of functional impairment, loss of mobility, and reduced quality of life.

Several studies on war-related injuries, including reports from Nigeria [37] and Cameroon [38], have emphasized that untreated nerve injuries result in long-term disability, especially in manual laborers and individuals with limited access to rehabilitation. The functional consequences of nerve injuries in conflict zones cannot be overstated, as they directly impact patients’ ability to work, perform daily activities, and regain independence.

Interestingly, a substantial number of patients in our study had undergone one or more previous surgeries (*n* = 97, 49.2%) before presenting for evaluation. This suggests that nerve injuries may have been overlooked or inadequately diagnosed during initial treatment, leading to sequential procedures rather than definitive repair. This highlights an urgent need to improve diagnostic accuracy for nerve injuries in low-resource, conflict settings.

Rapid and reliable diagnostic techniques, such as bedside nerve conduction tests or portable ultrasound for nerve assessment, could help ensure earlier recognition of nerve injuries and prevent unnecessary delays in surgical intervention. In settings where advanced diagnostic tools are unavailable, simple clinical assessment algorithms—such as the British Medical Research Council grading for motor function—should be implemented to guide treatment decisions [39, 40].

### The need for specialized surgical teams in conflict settings

The distribution of injury patterns and required surgical interventions in this study further emphasizes the necessity of specialized surgical expertise in war zones. Our findings indicate that bullet injuries were more often associated with functional deficits and movement restrictions, while blast injuries led to a higher frequency of fractures, soft tissue wounds, and contractures. These differences in trauma patterns directly translate into distinct surgical needs, requiring expertise in nerve surgery, reconstructive procedures, and limb salvage techniques. Based on the patterns of injuries and planned procedures observed in this study, we suggest that specialised surgical teams deployed to similar conflict settings should at minimum include a surgeon with experience in reconstructive and peripheral nerve surgery, an orthopaedic or trauma surgeon, an anaesthetist, and operating theatre and ward nurses with trauma and perioperative care expertise. Where feasible, access to physiotherapists or occupational therapists with experience in post-traumatic rehabilitation is highly desirable. In terms of technical capacity, teams would ideally be equipped to perform tendon transfers and lengthening procedures, contracture release and scar revision, fracture stabilisation, and basic peripheral nerve procedures, using appropriate instruments, implants, splinting materials and rehabilitation equipment.

Given the limited availability of reconstructive and hand surgeons in conflict settings, short-term deployments of specialized surgical teams could provide targeted interventions, significantly improving outcomes. Reports from Médecins Sans Frontières (MSF) missions in Iraq and Afghanistan have demonstrated that focused, high-impact surgical teams—including plastic surgeons and hand surgeons—can effectively manage war-related injuries, especially if deployed early.

However, while specialized teams could greatly improve acute care, their effectiveness is dependent on timely access to injured patients. Our data confirm that delayed access to surgical care remains a primary challenge, and any deployment of surgical teams must be coupled with strategies to ensure access during active conflict situations.

**Fig. 7:**
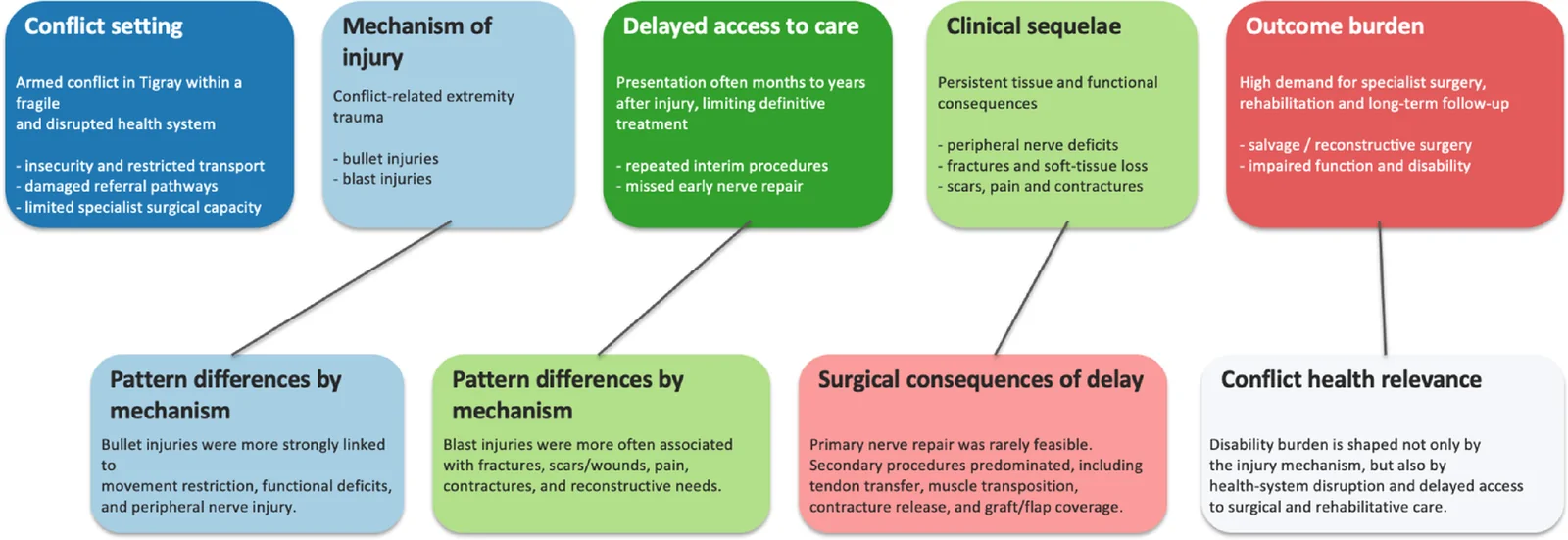
**Conceptual pathway linking conflict-related extremity trauma, delayed surgical care, and long-term disability in Tigray, Ethiopia.** The figure summarizes how bullet and blast injuries occurring in a disrupted conflict-affected health system may lead to delayed presentation, missed opportunities for early definitive treatment, persistent clinical sequelae—including peripheral nerve deficits, fractures, soft tissue loss, scars, pain, and contractures—and a high need for secondary reconstructive procedures such as tendon transfer, muscle transposition, contracture release, and graft or flap coverage. It is intended as an interpretive summary of the present findings rather than a tested causal model

Taken together, our findings can be conceptualized as a pathway in which conflict-related bullet and blast injuries occur in the context of health-system disruption, resulting in delayed access to definitive surgical care and, ultimately, long-term functional impairment (Fig. 7). This framework emphasizes that the disability burden observed in our cohort was shaped not only by the initial mechanism of injury, but also by prolonged delays in presentation, missed opportunities for early nerve repair, and limited access to specialist reconstructive and rehabilitative services.

## Conclusion

This study provides a comprehensive analysis of conflict-related injuries in Mek’ele, Ethiopia, focusing on gunshot and blast trauma. Our findings highlight distinct differences in injury patterns, surgical treatment needs, and long-term functional outcomes. Bullet injuries were more frequently associated with movement restrictions, functional deficits, and peripheral nerve damage, whereas blast injuries were linked to fractures, scars, and chronic pain. The high prevalence of peripheral nerve injuries, particularly in bullet-related trauma, underscores the need for early surgical intervention and specialized reconstructive expertise to optimize functional recovery.

A critical observation was the delayed access to surgical care, with most patients presenting months to years after their initial injury. This delay severely limited the feasibility of primary nerve repair and reconstructive procedures, necessitating secondary salvage surgeries such as tendon transfers. These findings emphasize the urgent need to improve trauma care accessibility and infrastructure in conflict-affected regions. Strengthening early intervention strategies, deploying specialized surgical teams, and implementing rapid diagnostic tools could significantly improve outcomes for war-injured patients.

Ultimately, our study underscores the importance of tailored surgical and rehabilitation strategies in conflict settings. As explosive and firearm-related injuries continue to impact civilian populations, particularly in sub-Saharan Africa, a coordinated effort is needed to enhance acute trauma management, long-term rehabilitation, and access to specialized surgical care. Addressing these challenges is crucial in mitigating disability and improving the quality of life for survivors of war-related injuries.

## Limitations

Despite the valuable insights provided by this study, several limitations must be acknowledged. As a single-center study conducted in Mek’ele, Ethiopia, the findings may not be fully generalizable to other conflict settings, where variations in healthcare infrastructure, access to surgical care, and injury mechanisms may influence outcomes. Additionally, although the analysis was based on routinely collected clinical data, certain variables relied on patient-reported histories, including previous surgeries and symptoms, introducing the possibility of recall bias. Selection bias must also be considered, as the study only includes patients who were able to reach medical care, potentially excluding those who succumbed to their injuries before reaching the hospital or those who had limited access to healthcare due to ongoing conflict. Another key limitation is the absence of a control group of patients with similar injuries in non-conflict settings, which could have provided valuable comparative insights into the specific challenges posed by war-related trauma. Despite these limitations, the study offers crucial data on conflict-related injuries in an understudied region. Future research should focus on multi-center studies, long-term follow-up assessments, and the integration of advanced diagnostic tools to refine our understanding of injury patterns and optimize treatment strategies in conflict settings.

## Data Availability

The datasets generated and analyzed during the current study are not publicly available due to concerns regarding participant confidentiality and the sensitive nature of conflict-related clinical data but are available from the corresponding author on reasonable request.

## References

[1] Heidelberg Institute for International Conflict Research (HIIK). Conflict Barometer 2023 [2024] [Available from: https://hiik.de/conflict-barometer/current-version/?lang=en. Accessed: 03.02.2025].

[2] Sharma P, Sharma A, Rao KR. The Changing Paradigm of Injuries and their Outcome in an International Conflict Zone. J Mar Med Soc. 2022;24(1):76–9.

[3] Mabry RL, Holcomb JB, Baker AM, Cloonan CC, Uhorchak JM, Perkins DE, et al. United States Army Rangers in Somalia: an analysis of combat casualties on an urban battlefield. J Trauma. 2000;49(3):515–28. discussion 28 – 9. 10.1097/00005373-200009000-00021 11003332

[4] Peleg K, Aharonson-Daniel L, Stein M, Michaelson M, Kluger Y, Simon D, et al. Gunshot and explosion injuries: characteristics, outcomes, and implications for care of terror-related injuries in Israel. Ann Surg. 2004;239(3):311–8. 10.1097/01.sla.0000114012.84732.be PMC1356227 15075646

[5] Benzinger TL, Brody D, Cardin S, Curley KC, Mintun MA, Mun SK, et al. Blast-related brain injury: imaging for clinical and research applications: report of the 2008 st. Louis workshop. J Neurotrauma. 2009;26(12):2127–44. 10.1089/neu.2009.0885 PMC2824226 19508154

[6] Champion HR, Holcomb JB, Young LA. Injuries from explosions: physics, biophysics, pathology, and required research focus. J Trauma. 2009;66(5):1468–77. discussion 77. 10.1097/TA.0b013e3181a27e7f 19430256

[7] Dilday J, Owattanapanich N, Benjamin ER, Biswas S, Shackelford S, Demetriades D. Injury Patterns and Treatment Strategies in Civilian and Military Isolated Abdominal Gunshot Wounds. World J Surg. 2023;47(11):2635–43. 10.1007/s00268-023-07132-z 37530783

[8] Muss TE, Hu S, Bauder AR, Lin IC. The Epidemiology, Management, and Outcomes of Civilian Gunshot Wounds to the Upper Extremity at an Urban Trauma Center. Plast Reconstr Surg Glob Open. 2024;12(4):e5753. 10.1097/GOX.0000000000005753 PMC11023610 38633511

[9] DePalma RG, Burris DG, Champion HR, Hodgson MJ. Blast injuries. N Engl J Med. 2005;352(13):1335–42. 10.1056/NEJMra042083 15800229

[10] Luria S, Rivkin G, Avitzour M, Liebergall M, Mintz Y, Mosheiff R. Comparative outcome of bomb explosion injuries versus high-powered gunshot injuries of the upper extremity in a civilian setting. Isr Med Assoc J. 2013;15(3):148–52. 23662376

[11] Asgedom AA, Etsedingl A, Hailemariam TT, Tequare MH, Hailu T, Tsegay AT, et al. Prevalence, causes and outcomes of war-related civilian injuries in Ethiopia’s war-torn Tigray region: a community-based descriptive study. BMC Res Notes. 2023;16(1):352. 10.1186/s13104-023-06640-4 PMC10683136 38012754

[12] World Health Organization (WHO). Building peace in fragile and conflict settings through health [2024] [Available from: https://www.who.int/activities/building-peace-in-fragile-and-conflict-settings-through-health. Accessed: 17.02.2025].

[13] Murray CJ, King G, Lopez AD, Tomijima N, Krug EG. Armed conflict as a public health problem. BMJ. 2002;324(7333):346–9. 10.1136/bmj.324.7333.346 PMC1122272 11834565

[14] Grasa R, Mateos O, Conflict. Peace and Security in Africa: An Assessment and New Questions after 50 Years of African Independence [2010] [Available from: https://ssrn.com/abstract=1884194. Accessed: 17.02.2025].

[15] Iordache SD, Gorski A, Nahas M, Feintuch L, Rahamimov N, Frenkel Rutenberg T. Treatment of Peripheral Nerve Injuries in Syria’s War Victims: Experience from a Northern Israeli Hospital. Isr Med Assoc J. 2021;23(5):279–85. 34024043

[16] Felder JM, Ducic I. Chronic Nerve Injuries and Delays in Surgical Treatment Negatively Impact Patient-reported Quality of Life. Plast Reconstr Surg Glob Open. 2021;9(5):e3570. 10.1097/GOX.0000000000003570 PMC8140770 34036022

[17] Statista. Median age in Africa 2000–2030. 2024.

[18] Kabiru CW, Izugbara CO, Beguy D. The health and wellbeing of young people in sub-Saharan Africa: an under-researched area? BMC Int Health Hum Rights. 2013;13:11. 10.1186/1472-698X-13-11 PMC3583739 23406522

[19] Mohamed AY, Ibrahim HS, Taskoparan H, Ibrahim YB. Epidemiological characteristics and comparative outcome of blast versus gunshot injuries of the extremities in Somalia. J Orthop Surg Res. 2023;18(1):44. 10.1186/s13018-023-03527-9 PMC9843833 36647060

[20] Human Sciences Research Council (HSRC). Young Men and the Construction of Masculinity in Sub-Saharan Africa: Implications for Participation in Violence. Human Sciences Research Council (HSRC); 2006.

[21] Amnesty International. Sub-Saharan Africa: The Devastating Impact of Conflicts Compounded. 2021.

[22] Dorward N. The urbanization of conflict? Patterns of armed conflict and protest in Africa. Afr Affairs. 2024;123(493):468–01. 10.1093/afraf/adae025.

[23] Chevalley K, Zimmerman J, Mittendorf A, Sennersten F, Dalman A, Frogh S, et al. Civilian pattern of injuries in armed conflicts - a systematic review. Scand J Trauma Resusc Emerg Med. 2024;32(1):125. 10.1186/s13049-024-01299-7 PMC11616296 39633489

[24] Maitland L, Middleton L, Veen H, Harrison DJ, Baden J, Hettiaratchy S. Analysis of 983 civilian blast and ballistic casualties and the generation of a template of injury burden: An observational study. EClinicalMedicine. 2022;54:101676. 10.1016/j.eclinm.2022.101676 PMC9530474 36204004

[25] Odatuwa-Omagbemi DO, Otene CI, Enemudo RET, Imonijevwe ES, Sefia TE. Gunshot injuries: experience in a tertiary health facility in the Niger Delta Region of Nigeria. Pan Afr Med J. 2022;43:133. 10.11604/pamj.2022.43.133.31587 PMC9898772 36762158

[26] Kelly JF, Ritenour AE, McLaughlin DF, Bagg KA, Apodaca AN, Mallak CT, et al. Injury severity and causes of death from Operation Iraqi Freedom and Operation Enduring Freedom: 2003–2004 versus 2006. J Trauma. 2008;64(2 Suppl):S21-6(discussion S 26-27). 10.1097/TA.0b013e318160b9fb 18376168

[27] Ramasamy A, Hill AM, Masouros S, Gibb I, Bull AM, Clasper JC. Blast-related fracture patterns: a forensic biomechanical approach. J R Soc Interface. 2011;8(58):689–98. 10.1098/rsif.2010.0476 PMC3061094 21123255

[28] Mokake NDM, Atabong BM, Ekani EM, Yawa N, Nana N, Theophile N, et al. Clinical Characteristics, Treatment and Outcome of Gunshot Injuries in South West Region of Cameroon. Clin Surg. 2024;10:01–10.

[29] Mabry RL, Apodaca A, Penrod J, Orman JA, Gerhardt RT, Dorlac WC. Impact of critical care-trained flight paramedics on casualty survival during helicopter evacuation in the current war in Afghanistan. J Trauma Acute Care Surg. 2012;73(2 Suppl 1):S32–7. 10.1097/TA.0b013e3182606001 22847091

[30] Alsalqawi A, Villar R. Patterns of Injury in a Gaza War Hospital. medRxiv [Prepring]. 2024. 10.1101/2024.06.27.24309570.

[31] UN Women. War on women: proportion of women killed in armed conflicts doubles in 2023. New York: UN Women; 2024 Oct 22 [cited 2026 Apr 17]. Available from: https://www.unwomen.org/en/news-stories/press-release/2024/10/war-on-women-women-killed-in-armed-conflicts-double-in-2023.

[32] Schneider F, Neidhardt M. Besonderheiten und Versorgung penetrierender Verletzungen am Beispiel von Schuss- und Explosionsopfern ohne ballistischen Körperschutz in Afghanistan (2009–2013). Wehrmedizin und Wehrpharmazie. 2016;4:12–7.

[33] Moustafa MK, Al-Hajj S, El-Hechi M, El Moheb M, Chamseddine Z, Kaafarani HMA. The Burden of Surgical Disease and Access to Care in a Vulnerable Syrian Refugee Population in Lebanon. World J Surg. 2021;45(10):3019–26. 10.1007/s00268-021-06242-w PMC8313117 34312694

[34] Smith ER, Concepcion TL, Shrime M, Niemeier K, Mohamed M, Dahir S, et al. Waiting Too Long: The Contribution of Delayed Surgical Access to Pediatric Disease Burden in Somaliland. World J Surg. 2020;44(3):656–64. 10.1007/s00268-019-05239-w 31654200

[35] Braga Silva J, Becker AS, Leal BLM, Busnello CV. Advances of Direct Peripheral Nerve Repair Techniques: Do We Already Have Enough Scientific Evidence? Indian J Orthop. 2023;57(2):189–202. 10.1007/s43465-022-00791-5 PMC9880092 36777121

[36] Schwartz HG, Parker JM. Early nerve and bone repair in war wounds. J Neurosurg. 1945;2:510–5. 10.3171/jns.1945.2.6.0510 21007727

[37] Ogwumike OO, Adeniyi AF, Badaru U, Onimisi JO. Profile of children with new-born brachial plexus palsy managed in a tertiary hospital in Ibadan, Nigeria. Niger J Physiol Sci. 2014;29(1):1–5. 26196558

[38] Nguembu S, Kenfack YJ, Sadler S, Zolo Y, Figuim B, Sebopelo LA, et al. Factors Associated with Adverse Outcomes in Cameroonian Patients with Traumatic Brain Injury: A Cross-Sectional Study. World Neurosurg. 2023;172:e62–7. 10.1016/j.wneu.2022.11.136 36481439

[39] Naqvi U, Sherman AL. Muscle Strength Grading. StatPearls. Treasure Island (FL) ineligible companies. Disclosure: Andrew Sherman declares no relevant financial relationships with ineligible companies.2025.

[40] Medical Research Council. MRC Muscle Scale United Kingdom. UK Research and Innovation (UKRI); [2023] [updated 2023. Available from: https://www.ukri.org/councils/mrc/facilities-and-resources/find-an-mrc-facility-or-resource/mrc-muscle-scale/. Accessed: 12.02.2025].

